# Effect of etelcalcetide on cardiac hypertrophy in hemodialysis patients: a randomized controlled trial (ETECAR-HD)

**DOI:** 10.1186/s13063-019-3707-7

**Published:** 2019-10-24

**Authors:** Katharina Dörr, Michael Kammer, Roman Reindl-Schwaighofer, Matthias Lorenz, Christian Loewe, Rodrig Marculescu, Reinhold Erben, Rainer Oberbauer

**Affiliations:** 1 Department of Nephrology, Medical University of Vienna, Spitalgasse 23, 1090 Vienna, Austria; 2 Center for Medical Statistics, Informatics and Intelligent Systems (CeMSIIS), Section for Clinical Biometrics, Medical University of Vienna, Spitalgasse 23, 1090 Vienna, Austria; 3 Vienna Dialysis Center, Kapellenweg 37, 1220 Vienna, Austria; 4 Division of Cardiovascular and Interventional Radiology, Department of Bioimaging and Image-Guided Intervention, Medical University of Vienna, Spitalgasse 23, 1090 Vienna, Austria; 5 Laboratory Medicine, Medical University of Vienna, Spitalgasse 23, 1090 Vienna, Austria; 6 Physiology, Pathophysiology, and Experimental Endocrinology, VetMed Vienna, Veterinärplatz 1, Vienna, Austria

**Keywords:** Hemodialysis, Left ventricular hypertrophy, Secondary hyperparathyroidism, FGF23, Etelcalcetide, Alfacalcidol

## Abstract

**Background:**

Fibroblast growth factor 23 (FGF23) is associated with left ventricular hypertrophy (LVH) in patients with chronic kidney disease, and calcimimetic therapy reduces plasma concentrations of FGF23. It remains unknown whether treatment with the calcimimetic etelcalcetide (ETL) reduces LVH in patients on hemodialysis.

**Methods/design:**

This single-blinded randomized trial of 12 months duration will test the effects of ETL compared with alfacalcidol on LVH and cardiac fibrosis in maintenance hemodialysis patients with secondary hyperparathyroidism. Both treatment regimens will be titrated to equally suppress secondary hyperparathyroidism while alfacalcidol treatment causes an increase and ETL a decrease in FGF23, respectively.

Patients treated thrice weekly with hemodialysis for ≥ 3 months and ≤ 3 years with parathyroid hormone levels ≥ 300 pg/ml and LVH will be enrolled in the study.

The primary study endpoint is change from baseline to 12 months in left ventricular mass index (LVMI; g/m^2^) measured by cardiac magnetic resonance imaging. Sample size calculations showed that 62 randomized patients will be necessary to detect a difference in LVMI of at least 20 g/m^2^ between the two groups at 12 months. Due to the strong association of volume overload and LVH, randomization will be stratified by residual kidney function, and regular body composition monitoring will be performed to control the volume status of patients.

Study medication will be administered intravenously by the dialysis nurses after every hemodialysis session, thus omitting adherence issues.

Secondary study endpoints are cardiac parameters measured by echocardiography, biomarker concentrations of bone metabolism (FGF23, vitamin D, parathyroid hormone, calcium, phosphate, s-Klotho), cardiac markers (pro-brain natriuretic peptide, pre- and postdialysis troponin T) and metabolites of the renin–angiotensin–aldosterone cascade (angiotensin I (Ang I), Ang II, Ang-(1–7), Ang-(1–5), Ang-(1–9), and aldosterone).

**Discussion:**

The causal inference and pathophysiology of LVH regression by FGF23 reduction using calcimimetic treatment has not yet been shown. This intervention study has the potential to discover a new strategy for the treatment of cardiac hypertrophy and fibrosis in patients on maintenance hemodialysis. It might be speculated that successful treatment of cardiac morphology will also reduce the risk of cardiac death in this population.

**Trial registration:**

European Clinical Trials Database, EudraCT number 2017-000222-35; ClinicalTrials.gov, NCT03182699. Registered on

## Background

Patients with chronic kidney disease (CKD) develop left ventricular hypertrophy (LVH) and cardiac fibrosis which contributes to congestive heart failure, diastolic dysfunction, arrhythmia and sudden death [1–3]. The majority of patients with terminal renal failure treated by dialysis exhibit LVH and have a dramatically increased risk of sudden cardiac death [4].

The main drivers of cardiac remodeling in hemodialysis patients are chronic volume overload, intradialytic weight gain and hemodynamic fluctuations during hemodialysis treatment [5, 6]. Additional factors include elevated fibroblast growth factor 23 (FGF23) levels in CKD and dialysis patients and angiotensin II (Ang II)-mediated cardiac remodeling [7, 8]. Circulating concentrations of FGF23 increase progressively as the glomerular filtration rate declines, beginning as early as CKD stage 3b [9–14]. The biological effects of FGF23 are mediated through a receptor complex consisting of FGF receptors (FGFRs) and of the co-receptor α-Klotho, which enables proper FGF23 signaling in target tissues such as the kidney [15].

The left ventricular mass index (LVMI) rises with increasing FGF23 as does the prevalence of eccentric and concentric hypertrophy [2]. The pathophysiological mechanism by which FGF23 may cause LVH is still not well understood and two potentially synergistic hypotheses are discussed in the scientific community.

Wolf et al. showed a direct effect of FGF23 on myocardial hypertrophy. FGF23 treatment of isolated neonatal mouse cardiomyocytes caused an increase in surface area and an activation of pro-hypertrophic gene programs that was independent of Klotho and mediated through FGFR4 [1, 2, 16].

Andrukhova et al. proposed a complementary concept by stating that FGF23-induced Na^+^ and Ca^2+^ retention, volume overload and hypertension are the most determinant factors underlying the pro-hypertrophic effects [17–19]. The investigators were able to show that a low-dose anti-FGF23 antibody treatment substantially ameliorated disease progression and left ventricular dysfunction by preventing the abovementioned volume overload and its consequences on the circulation (unpublished data). Additionally, they showed that the administration of chlorothiazide completely prevents FGF23-induced volume expansion and heart hypertrophy [17].

Recently, Slavic et al. provided evidence of increasing levels of FGF23 and Klotho in a mouse model with pressure overload-induced LVH. They identified aldosterone to be an important stimulator of bone FGF23 transcription following pressure overload [20].

The association of FGF23 and LVH via an activation of the renin–angiotensin–aldosterone system (RAAS) through suppression of angiotensin-converting enzyme 2 (ACE2), and therefore increasing its product Ang-(1–7), have been described previously [21–29]. An overactive RAAS has been linked to multiple pathological processes such as LVH and heart failure, and medications inhibiting the RAAS are capable of improving both [7, 8, 30–32].

The HEMO study investigated a cohort of 1340 hemodialysis patients and found that higher FGF23 levels were a predictor of cardiac events, infections and all-cause mortality [33]. Various studies, such as PARADIGM, demonstrated that the oral calcimimetic drug cinacalcet causes a reduction in the level of FGF23 of at least 30%, while the intake of vitamin D analogs causes an increase of over 40%. Both treatments cause similar modest reductions in parathyroid hormone (PTH) levels [34–37].

In this trial, the level of FGF23 will be modified by either the calcimimetic etelcalcetide (ETL) or alfacalcidol (ALFA) at a PTH clamp, and therefore will be able to test the causality of FGF23 reduction on cardiac hypertrophy and fibrosis.

## Methods/design

### Study design

In this randomized, controlled, single-blinded trial, we will study the effect of the calcimimetic drug ETL in comparison with the active vitamin D ALFA on LVH and cardiac fibrosis in hemodialysis patients with secondary hyperparathyroidism (sHPT).

The treatment will be administered intravenously by dialysis nurses in addition to conventional HPT therapy (phosphate binders, calcium supplementation) in 62 subjects for 12 months. LVH will be measured as LVMI by cardiac magnetic resonance imaging (cMRI). The inclusion and exclusion criteria for participants are listed in Table 1. Patients will be recruited from two hemodialysis centers of the Medical University of 144 Vienna with 160 prevalent patients and the Vienna Dialysis Center with 300 prevalent 145 patients. The present protocol follows the Standard Protocol Items: Recommendations for Interventional Trials (SPIRIT) guidelines and fulfills the SPIRIT checklist (see Additional file 1).

**Table 1:** Main inclusion and exclusion criteria

Main inclusion criteria	
Age ≥ 18 years	
Maintenance hemodialysis 3×/week for ≥ 3 months and ≤ 3 years	
sHPT defined by:	
• PTH ≥ 300 pg/mL and no prior calcimimetic drug, or	
• PTH ≥ 300 pg/mL after washout of vitamin D for 4 weeks	
• Patients after washout of cinacalcet for 4 weeks	
Serum calcium ≥ 2.08 mmol/L	
LVH ± cardiac fibrosis on echocardiography	
Optimal fluid composition (BCM measurement); pulmonary edema excluded (lung ultrasound)	
No substantial dose change of calcium supplements, phosphate binders, dialysate calcium, or active vitamin D for 4 weeks before screening	
Main exclusion criteria	
Unstable medical condition	
Significantly impaired LV systolic function or hemodynamically effective heart valve defects	
Anticipated parathyroidectomy	
Scheduled kidney transplant from a living donor	
Uncontrolled hyperphosphatemia	
Active participation in another clinical trial	
Sensitivity or intolerance to administered products	
Women who are pregnant or breast feeding	
Disorder compromising the ability to give informed consent and/or to comply with the study procedures	
Contraindications for MRI	

*BCM* body composition monitoring, *LV* left ventricular, *LVH* left ventricular hypertrophy, *MRI* magnetic resonance imaging, *PTH* parathyroid hormone, *sHPT* secondary hyperparathyroidism

### Screening, washout phase and randomization

**Fig. 1:**
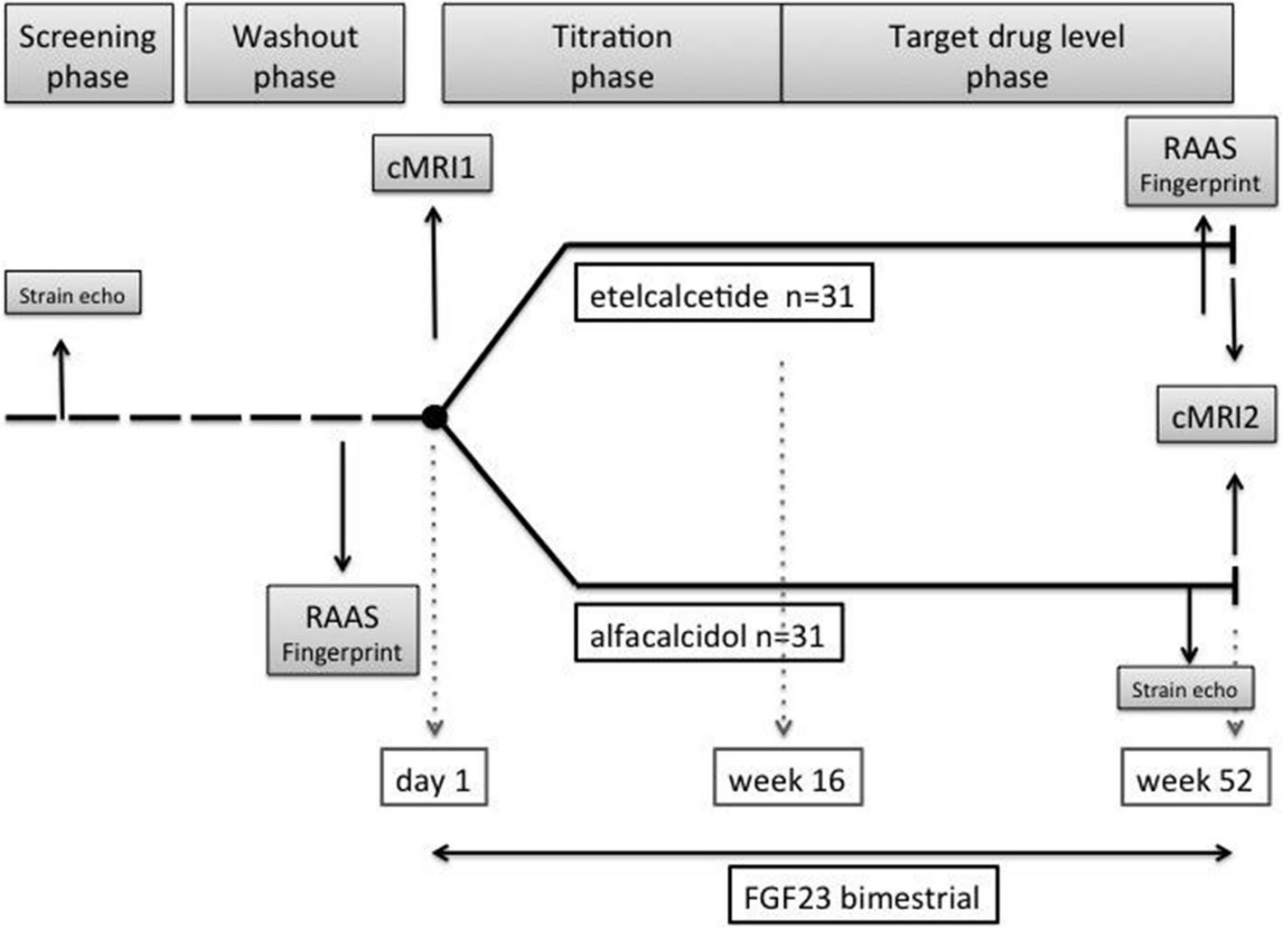
Study flow chart. cMRI cardiac magnetic resonance, Echo echocardiography, FGF23 fibroblast growth factor 23, RAAS renin–angiotensin–aldosterone system

**Fig. 2:**
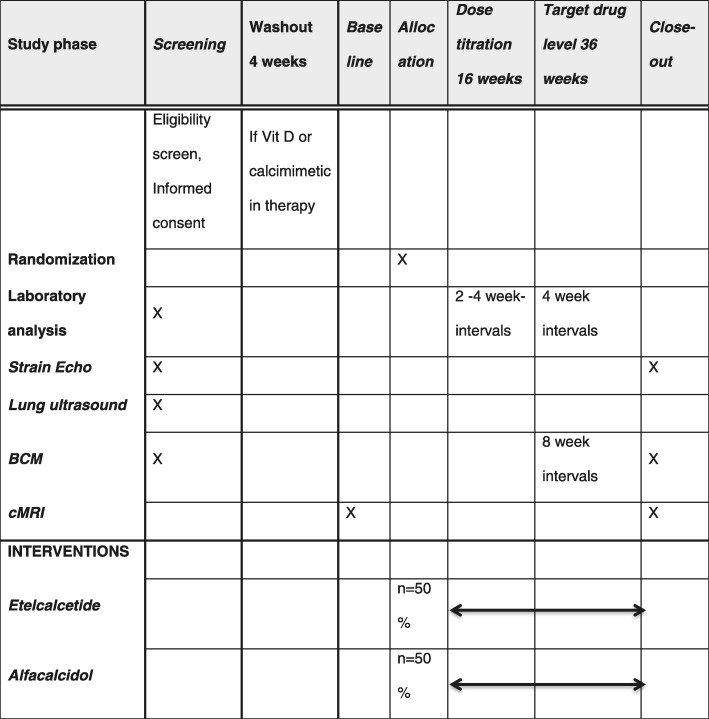
Study design. BCM body composition monitoring, cMRI cardiac magnetic resonance imaging, Echo echocardiography, Vit vitamin

The study flow chart and design are presented in Figs. 1 and 2, respectively. Following signed informed consent, patients will be screened for LVH (i.e., interventricular septum thickness ≥ 12 mm) and cardiac fibrosis using strain echocardiography. Volume status and fluid composition will be explored with the help of body composition monitoring (BCM) and lung ultrasound [38–41]. Only patients who are stable at their dry weight are eligible for enrollment to the study. All patients that are already being treated with a calcimimetic drug or vitamin D therapy will undergo a 4-week-long washout phase in which the treatment will be discontinued. Study participants who qualify for the study will be randomized at a 1:1 ratio to the ETL group or the ALFA group. Randomization will be performed by a computer algorithm (www.meduniwien.ac.at/randomizer/web) and will be stratified by residual kidney function (< 500 ml versus ≥ 500 ml urine per day) and the center where patients are recruited (Medical University of Vienna versus Vienna Dialysis Center). To ensure that comparison groups will be of approximately the same size and balanced in each center, a block randomization (block size of 4) will be used.

### Treatment phase

The treatment phase starts with a dose-titration phase of 16 weeks. Subjects will be considered for dose titration of the investigational product every 4 weeks. Dose adjustment will be based upon PTH values, serum electrolytes and safety assessment. Study visits will take place in 2-week intervals during the first 10 weeks of treatment followed by study visits every 4 weeks. The duration of the treatment phase is 12 months.

### Study endpoints

The primary endpoint is the change in LVMI (quantified in grams per meter squared) from baseline to 12 months between the ETL and ALFA groups as assessed by cMRI.

Secondary endpoints are the change in left atrial diameter (measured in millimeters), the change in LVMI and left atrial diameter progression (percent), the difference in cardiac fibrosis and fibrosis progression as measured with noncontrast T1 mapping (milliseconds) and differences in cardiac function (ejection fraction, measured as percent) as well as wall motion abnormalities (percent change) as measured by cMRI and strain echocardiography after 1-year treatment with either drug. Other secondary objectives include changes in serum levels of FGF23, s-Klotho, PTH, 25-hydroxyvitamin D (25(OH)D) and 1,25-dihydroxyvitamin D (1,25(OH)_2_D), phosphate, calcium, pro-brain natriuretic peptide (proBNP), pre- and postdialysis troponin T (TnT) and the metabolites of the RAAS cascade (Ang I, Ang II, Ang-(1–7), Ang-(1–5), Ang-(1–9), aldosterone) under either treatment as well as their association with the abovementioned cardiac changes.

### Outcome measurements

#### Cardiac MRI

Two cMRIs are planned for each patient. The baseline MRI will take place before randomization and the second MRI will take place after completing 12 months of treatment. Both will be carried on the dialysis-free day.

The cMRI will be analyzed by one radiologist blinded to the treatment allocation. Noncontrast cMRI will be carried out using a 1.5-Tesla MRI scanner (Siemens Avanto 1.5 T, Siemens, Erlangen, Germany). Axial black-blood imaging will be performed for visualization of cardiac anatomy. For the assessment of cardiac function, left ventricular muscle mass, and the visualization of possible wall motion abnormalities, multislice-multiphase cine imaging will be performed in the long horizontal axis as well as in the short axis view through the entire heart. The ejection fraction (in percent) of both the left and right ventricles will be calculated in a semiautomatic fashion using dedicated software (Siemens Argus) based on the short axis views. For the assessment of cardiac function, the end-diastolic and end-systolic volume (in milliliters) will be assessed in a semiautomatic fashion and the left ventricular muscle mass will be calculated [42]. The upper limit of normal left ventricular mass indexed for body surface area (LVM/BSA) values is considered to be 84.1 g/m^2^ for male and 76.4 g/m^2^ for female subjects [6].

For the detection of myocardial fibrosis, fat-suppressed T2-weighted edema-sensitive imaging will be performed. Noncontrast T1 mapping will be performed to detect diffuse fibrotic processes (T1 time is measured in milliseconds for global, septal and nonseptal times). The native myocardial T1 relaxation is a surrogate of myocardial fibrosis [43]. In hemodialysis patients the interventricular septum appears to be particularly prone to the development of fibrosis [44].

#### Strain echocardiography

Echocardiography for the evaluation of LVH will take place during screening as well as at the end of the treatment phase. Doppler imaging or two-dimensional speckle tracking echocardiography is used to measure strain and strain rate. With these techniques subclinical heart disease in fibrotic processes can be detected, with the predominant planes of strain that are initially affected mirroring the histological location of early fibrosis [45, 46]. Global longitudinal strain is measured as percent and correlates well with myocardial fibrosis [47]. The physician performing the examination will be blinded to the patient’s treatment assignment.

#### Body composition monitoring

BCM will be performed during screening and will be repeated at 2-month intervals. BCM measurements are based on bioimpedance spectroscopy. The measurements are fed into a model to measure overhydration of an individual [41]. Fluid overload assessed by BCM is expressed as an absolute value in liters or as a relative value as a percent [48]. It is a reproducible body fluid volume determination over a wide range of body compositions at different states of health and disease [40]. Only patients who achieve their optimal dry weight at the end of dialysis treatment and tolerate it well will be enrolled in the study. Should patients present themselves with hypervolemia as measured by BCM during the treatment phase, the dry weight will be adapted dependent on BCM results in accordance with clinical judgment and standard of care equally in both treatment groups.

#### Lung ultrasound

The assessment of extravascular lung water will take place as part of the screening procedures with the help of lung ultrasound, which can visualize lung edema and classify it semiquantitatively [38, 39, 49]. Only patients without signs of pulmonary edema will be enrolled in the study.

#### Laboratory analyses

Biochemical data will be collected prior to hemodialysis at baseline and periodically (e.g., intact PTH, calcium, phosphate, 25(OH)D, 1,25(OH)_2_D every 2 weeks during the first 10 weeks followed by measurements every 4 weeks; while intact FGF23, s-Klotho and pre- and postdialysis TnT will be measured at 8-week intervals). Furthermore, proBNP levels will be measured as a marker of body fluid volume every 8 weeks. Additionally, an RAAS fingerprint will be conducted at the start and at the end of the treatment phase [25, 28, 29, 50]. The RAAS fingerprint is a mass spectrometry-based quantification of angiotensin metabolites, which will be performed by a resident diagnostic service provider (Attoquant Diagnostics). Serum samples will be used to measure the following parameters: Ang I, Ang II, Ang-(1–7), Ang-(1–5), Ang-(1–9) and aldosterone.

Intact PTH, calcium and phosphate will be analyzed in serum samples using the Cobas assay (Roche; reference ranges: PTH 15–65 pg/ml, calcium 2.15–2.55 mmol/l and phosphate 0.81–1.45 mmol/l). Vitamin D will be measured using serum samples chemiluminescent immunoassays (DiaSorin; reference ranges: 1,25-(OH)_2_D 19.9–79.3 pg/ml and 25(OH)D 75–250 nmol/l). Ionized calcium will be measured during every dialysis session (using blood gas analysis (ABL 800 Flex, Drott)). Intact FGF23 will be analyzed in plasma samples using chemiluminescent immunoassays (DiaSorin; reference range: 23.2–95.4 pg/ml). TnT and proBNP will be measured from serum samples using Cobas electrochemiluminescence immunoassays (Roche; reference ranges: TnT 0–14 ng/L and proBNP 0–125 pg/ml).

**Fig. 3:**
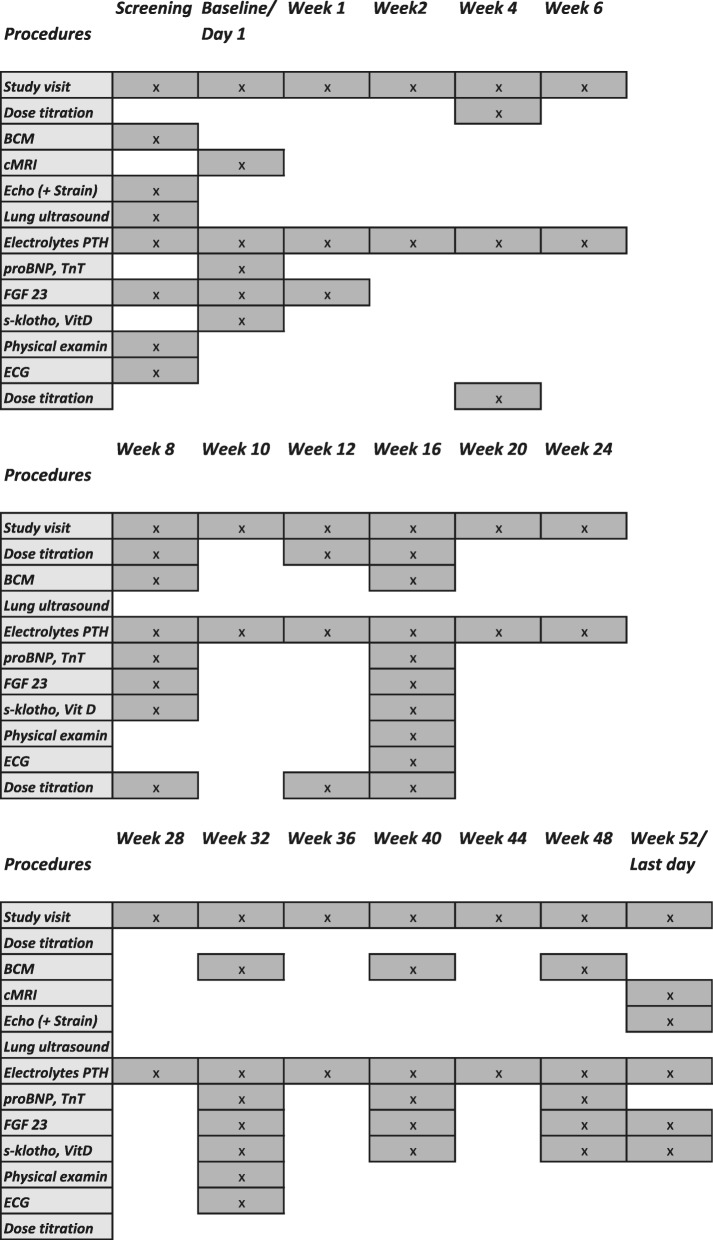
Study synopsis

The timeline of the planned procedures, study visits and scheduled dose titrations is shown in Fig. 3.

### Investigational products

The pharmacodynamics of ETL and cinacalcet are similar. They both cause rapid, dose-dependent decreases in circulating levels of PTH, FGF23, calcium and phosphorus in CKD patients.

A single intravenous dose of ETL can lower serum levels of PTH for up to 72 h in patients on hemodialysis. FGF23 levels decrease by over 30% at 24 h after a single 10-mg dose of ETL, while little or no effect is shown on 1,25(OH)_2_D levels in a study conducted by Martin et al. [51]. The most frequent treatment-emergent adverse event is a decrease in blood calcium [52]. ETL dosage should be between 2.5 mg and 15 mg three times a week. The starting dose is 5 mg three times a week. To achieve a target value of PTH (100–300 pg/ml), the dosage will be adapted every 4 weeks in steps of 2.5 or 5 mg during the titration phase. Serum calcium will be measured at every dialysis session. Target levels of serum calcium corrected for serum albumin are ≥ 2.08 mmol/l.

ALFA is an analogue of vitamin D3. ALFA can decrease PTH levels by ≥ 30% and increase FGF23 levels threefold [53]. In general, ALFA is a safe and well-tolerated established treatment for sHPT.

The starting dose is 1 μg, administered as an intravenous bolus three times a week at the end of hemodialysis. ALFA dosage should be at least 0.5 μg three times a week with no maximal dose. Titration will be performed in 0.5- to 1-μg steps at 4-week intervals, depending on PTH values and serum calcium and phosphate levels. The target value of PTH is equivalent to the ETL group. Serum calcium corrected for serum albumin should be no higher than 2.55 mmol/l and serum phosphate levels should be below 2.5 mmol/l.

The goal is to achieve a similar reduction in PTH in both study groups while FGF23 is elevated in the ALFA arm and suppressed in the ETL arm in order to analyze the causality of FGF23 reduction on LVH and fibrosis.

However, it is likely that the levels of PTH will vary, simply due to the different pharmacodynamics of the two drugs. Even though the dose of the study medication can be changed during the drug titration period as well as later on when necessary in order to reach target PTH levels, these adaptations are often limited by serum calcium and phosphate levels.

#### Other HPT treatments

Cinacalcet treatment as well as oral and intravenous vitamin D therapy will be discontinued during the washout phase of 4 weeks. Phosphate binder therapy can be continued and will be adapted depending on serum electrolytes during the treatment phase. There are no restrictions on calcium supplements, the dialysate calcium concentration, or the type or dose of phosphate binders prescribed. Participants randomized to ETL can receive additional vitamin D analogs as a rescue therapy only when the investigator thinks that it is necessary to protect participant safety.

### Data Safety Monitoring Board

An independent Data Safety Monitoring Board of the Medical University of Vienna will be convened to assess the safety of treatment as well as the superiority of one treatment over the other [54, 55]. Interim analysis will be performed by the board after the completed follow-up of 10 cases in each treatment group (one-third of the planned study population). The Lan and DeMets alpha spending method using O’Brien–Fleming type boundaries will be applied and the trial will be stopped if *p* < 0.000207 [56].

### Quality control and quality assurance

The study monitor will contact and visit the investigator regularly and will be allowed, on request, to have access to all source documents needed to verify the entries in the electronic documentation and other study-related documents provided that subject confidentiality is maintained in agreement with local regulations. It will be the monitor’s responsibility to inspect the electronic case report forms at regular intervals throughout the study to verify the adherence to the study protocol and the completeness, consistency and accuracy of the data being entered. The monitoring standards require full verification for the presence of informed consent, adherence to the inclusion/exclusion criteria, documentation of serious adverse events (AEs)/serious adverse device effects and the recording of the main efficacy, safety, and tolerability endpoints. At least three monitoring visits are scheduled. The monitor will be working according to standard operating procedures and will provide a monitoring report after each visit for the sponsor and the investigator.

### Safety evaluation and reporting of adverse events

The investigators ensure that adequate medical care is provided in any clinical situation, including emergencies. All AEs observed by the investigator or reported by subjects are to be properly captured in the subjects’ medical records. This collection period will be from the time of the first dose of the investigational product to 30 days after the last dose.

It will be left to the investigator’s clinical judgment to determine whether an AE is related and of sufficient severity to require the subject’s removal from treatment. As defined by the International Conference on Harmonization guidelines and World Health Organization Good Clinical Practice guidelines, serious AEs are events that result in patient death, are life-threatening, require or prolong hospital stay, cause persistent or significant disability or incapacity, result in congenital anomaly or birth defect, or necessitate specific interventions. Events that are suspected unexpected serious adverse reactions (SUSARs) will be reported to the responsible ethics committee — the European Medicines Agency via the Clinical Trials Coordination Center of the Medical University of Vienna. Fatal SUSARs will be reported as soon as possible, but at the latest within 7 days and nonfatal SUSARs within 15 days.

### Statistical methods

Data will be described as means and standard deviation or medians and interquartile range for continuous symmetric and skewed variables, respectively. Distributions of the analyzed parameters will be visualized by boxplots and histograms.

The primary endpoint (change in LVMI from baseline to 12 months) will be analyzed by the analysis of covariance. The main variable in the model to be tested will be treatment group, which represents the treatment effect on change in LVMI 1 year after baseline between the two treatments. Baseline LVMI for each patient will be used as a covariate in the model and the interaction between treatment group and baseline LVMI will be included. Furthermore, to account for stratification during randomization, the stratification factors will also be included in the model. The secondary endpoints (changes in FGF23, s-Klotho, PTH, 25(OH)D, 1,25(OH)_2_D, proBNP, pre- and postdialysis TnT and RAAS metabolites) will be analyzed analogously. All analyses will be conducted according to the intention-to-treat principle. Two-sided *p* values lower than 0.05 will indicate statistical significance.

### Sample size calculation

On the assumption, based on published data, that the mean LVM/BSA of hemodialysis patients determined by cMRI is 100 g/m^2^ with a standard deviation of 25 g/m^2^ [42] and an expected treatment effect of delta LVMI of 20 g/m^2^, 25 patients are needed per group to detect this difference with 80% power using a two-sample *t* test at an alpha level of 0.05. Assuming 10% attrition (drop out/loss to follow-up) within 1 year of follow-up, the final sample size in the intention-to-treat analysis will be 31 patients.

Patients receiving a renal transplant as well as those who become pregnant (which is unlikely due to the significantly reduced fertility of women under dialysis) will drop out of the study.

### Study registration

The study was approved by the Austrian regulatory authority (Federal Office for Safety in Health Care, Austrian Agency for Health and Food Safety, AGES reference number 10087746) and was registered in the European Clinical Trials database (EudraCT, 2017–000222-35) and in a public clinical trial database (ClinicalTrials.gov, NCT03182699).

## Discussion

In our proposed trial we will provide novel insights into the extent of FGF23-mediated cardiac remodeling in patients on chronic hemodialysis. We specifically hypothesize that treatment with ETL ameliorates pathological changes in the cardiac structure of dialysis patients with sHPT by suppression of systemic FGF23 levels.

The EVOLVE study investigated the effect of lowering FGF23 with the use of cinacalcet on cardiovascular mortality in 3883 hemodialysis patients with sHPT. They were able to show that a reduction in FGF23 of ≥ 30% after 20 weeks of therapy showed a trend towards a decrease in cardiovascular mortality, sudden cardiac death and heart failure [35, 57].

A small study conducted by Choi et al. described a significant reduction in LVMI and a significantly improved diastolic function, measured by echocardiography, in 12 hemodialysis patients treated with cinacalcet [58].

In our proposed trial we make use of the deviant influence of ETL versus ALFA on the serum levels of FGF23 since, as mentioned previously, calcimimetic drugs decrease FGF23 while vitamin D increases it. Consequently, we generated a human model to study the influence of changing serum FGF23 levels on cardiac structure using approved medication for sHPT. We established PTH target values of 100–300 pg/ml considering the Kidney Disease: Improving Global Outcomes guidelines in order to demonstrate the effect of FGF23 independent of PTH. Study medication will be provided intravenously, allowing a very consistent delivery of the drug. One of the most important advantages of the intravenous treatment is the elimination of possible noncompliance. Patient adherence to oral cinacalcet therapy is known to be very low [59]. Another major advantage of this study when comparing it to the trial by Choi et al. is the use of cMRI as the diagnostic tool for the quantification of left ventricular mass, lowering the inter- and intraobserver variability known from using echocardiography. cMRI provides accurate anatomic information that is in excellent agreement with autopsy results [60, 61]. It is also able to detect LVH in patients with seemingly normal echocardiographic results due to a geometric assumption-free quantification of left ventricular mass [62, 63].

Based on the strong association of volume overload with CKD progression and adverse cardiac outcome we will perform a stratified randomization procedure to ensure an equal distribution of dialysis patients with residual renal function (i.e., ≥ 500 ml urine/day) and those without (< 500 ml urine/day) in both treatment groups [64]. Additionally, only patients reaching their individual optimal dry weight will be allowed to participate in the study.

This trial is designed to treat patients with either study medication for 12 months. It can be argued that this amount of time is too short to reproduce a measurable change in cardiac structure. It is important to consider that it takes a certain amount of time to develop sHPT under dialysis and to reach a severity requiring intravenous treatment. Additionally, in Austria the median time spent on the waiting list for renal transplantation is around 3 years, not to mention the high mortality of patients under dialysis. Consequently, in order to avoid a high drop-out as well as out-of-feasibility reasons, we decided this precise follow-up time period.

The diagnosis of LVH prior to enrollment in the study poses a certain difficulty regarding the accuracy of echocardiographic quantification of LVH. However, since each patient serves as his or her own control, the progression of left ventricular mass can be demonstrated during the course of the 12 months of treatment.

This trial is designed to visualize changes in cardiac muscle mass and fibrosis as a result of modified FGF23 levels which might be causal to the improved cardiovascular outcomes under lower FGF23 described in the EVOLVE study. If our study proves that ETL can effectively ameliorate LVH and cardiac fibrosis trough a suppression of FGF23, it may potentially provide a valuable basis for a pharmaceutical target aiming at reducing the high rate of cardiac death in patients under maintenance hemodialysis.

## Trial status

This is Protocol version 1.0, 28 May 2019. Recruitment of study patients started in October 2017 and enrollment is estimated to be complete as of November 2019.

## Supplementary information

**Additional file 1.** SPIRIT 2013 Checklist: Recommended items to address in a clinical trial protocol and related documents.

## Data Availability

The data that support the findings of this study will be available from the corresponding author upon reasonable request.

## Abbreviations

1,25(OH)_2_D: 1,25-Dihydroxyvitamin D
25(OH)D: 25-Hydroxyvitamin D
ACE: Angiotensin-converting enzyme
AE: Adverse event
ALFA: Alfacalcidol
Ang: Angiotensin
BCM: Body composition monitoring
BNP: Brain natriuretic peptide
CKD: Chronic kidney disease
cMRI: Cardiac magnetic resonance imaging
ETL: Etelcalcetide
FGF: Fibroblast growth factor
FGFR: Fibroblast growth factor receptor
LVH: Left ventricular hypertrophy
LVM/BSA: Left ventricular mass indexed for body surface area
LVMI: Left ventricular mass index
PTH: Parathyroid hormone
RAAS: Renin–angiotensin–aldosterone system
sHPT: Secondary hyperparathyroidism
SUSAR: Suspected unexpected serious adverse reaction
TnT: Troponin T
