# Supplementary material for: Effect of etelcalcetide on cardiac hypertrophy in hemodialysis patients: a randomized controlled trial (ETECAR-HD)
Source: Trials. 2019 Oct 24;20:601. doi: 10.1186/s13063-019-3707-7 (PMC6813957; doi:10.1186/s13063-019-3707-7)
Supplement: Supplementary file 1 — Additional file 1. SPIRIT 2013 Checklist: Recommended items to address in a clinical trial protocol and related documents. [file 13063_2019_3707_MOESM1_ESM.pdf]

STANDARD PROTOCOL ITEMS: RECOMMENDATIONS FOR INTERVENTIONAL TRIALS

SPIRIT 2013 Checklist: Recommended items to address in a clinical trial protocol and related documents*

Section/item	Item	Study protocol	Page in manuscript
**Administrative information**			
Title	1	**E**ffect of e**tel**calcetide on **car**diac **h**ypertrophy in hemo**d**ialysis patients – a randomized controlled trial (ETECAR-HD)	1
Trial registration	2a	Austrian regulatory authority (Federal Office for Safety in Health Care, Austrian Agency for Health and Food Safety, AGES reference number: 10087746)<br><br>European Clinical Trials Database (EudraCT number 2017-000222-35)<br><br>Public clinical trial database (ClinicalTrials.gov ID: NCT03182699).	2
Protocol version	3	1.0, 28.May. 2019	16
Funding	4	Funding for this study was provided by the Vienna General Hospital (Amtsforschung) and an unrestricted grant from Amgen (reference# 20167811).	17

Roles and responsibilities	5a	Katharina Dörr - Department of Nephrology, Medical University of Vienna, Vienna, Austria: Design of the study, draft of manuscript<br>Rainer Oberbauer - Department of Nephrology, Medical University of Vienna, Vienna, Austria: Design of the study, draft of manuscript<br>Roman Reindl-Schwaighofer - Department of Nephrology, Medical University of Vienna, Vienna, Austria: Design of the study, draft of manuscript<br>Michael Kammer - Center for Medical Statistics, Informatics and Intelligent Systems (CeMSIIS), Section for Clinical Biometrics, Medical University of Vienna, Vienna, Austria: statistical planning of the study,<br>Matthias Lorenz - Vienna Dialysis Center, Vienna, Austria: provided feasibility expertise and data quality control<br>Loewe Christian - Department of Radiology, Medical University of Vienna, Vienna, Austria: performes and investigates cardiac MRIs<br>Rodrig Marculescu - Laboratory Medicine, Medical University of Vienna, Austria: performes most laboratory measurements and contributes to data quality control	17
	5b	Univ.Prof.Dr. Rainer Oberbauer	
	5c	Univ.Prof.Dr. Rainer Oberbauer is the sponsor of the study. He is the guarantor of this work and, as such, has full access to all the data in the study and takes responsibility for the integrity of the data and the accuracy of the data analysis. Together with K.D. he designed the study and was responsible for the interpretation of data, management of the study, writing of the report and decision to submit the report for publication.<br><br>Amgen provided an unrestricted grant which is used for materials and personnel costs (study nurses). Amgen looked over the study design but has no role in management, analysis, interpretation of data and does not decide on the submission of the report for publication.	
	5d	K.D. is the main manager of the trial and responsible for analysis and interpretation of data as well as writing of the report. She is the main coordinator in both study centers together with R.O. and M.L.<br><br>An independent data safety monitoring board (DSMB) of the Medical University of Vienna is convened to assess the safety of treatment as well as the non-superiority of one treatment over the other	

Introduction

Background and rationale	6a	FGF23 is associated with left ventricular hypertrophy (LVH) in patients with chronic kidney disease and calcimimetic therapy reduces plasma concentrations of FGF23. The majority of patients with terminal renal failure treated by dialysis exhibit LVH and have a dramatically increased risk of sudden cardiac death.<br>The EVOLVE study was able to show that a reduction of FGF23 after 20 weeks of cinacalcet therapy showed a trend towards a decrease in cardiovascular mortality, sudden cardiac death and heart failure<br>Wolf et al. showed a direct effect of FGF23 on mouse cardiomyocytes leading to hypertrophy.<br>The HEMO study found that higher FGF23 levels in hemodialysis patients were a predictor of cardiac events, infections and all-cause mortality<br>The aim of the present trial is the analysis of the causal inference and pathophysiology of LVH regression by FGF23 reduction by calcimimetic treatment. Study patients will receive medication approved for sHPT treatment. The diagnostic procedures are non invasive and harmless.	4-6
	6b	Subjects will either receive Etelcalcetide or Alfacalcidol in order to achieve a similar reduction in PTH in both study groups while FGF23 is elevated in the Vitamin D arm and suppressed in the Etelcalcetide arm in order to analyze the causality of FGF23 reduction on LVH and fibrosis.	
Objectives	7	We specifically hypothesize that treatment with Etelcalcetide ameliorates pathological changes in cardiac structure (Left ventricular mass and fibrosis) of dialysis patients with secondary hyperparathyroidism (sHPT) by suppression of systemic FGF23 levels.	8
Trial design	8	It is a controlled, single blinded trial where patients are randomized to one of two treatment groups.	6-14

Methods: Participants, interventions, and outcomes

Study setting	9	Patients will be recruited from two hemodialysis centers of the Medical University of Vienna and the Vienna Dialysis Center.	6

Eligibility criteria	10	Main inclusion criteria: Age ≥ 18 years; Maintenance hemodialysis 3x/week for ≥ 3 months and ≤3 years; sHPT defined by (PTH ≥300 pg/mL and no prior calcimimetic drug, or PTH ≥300 pg/mL after washout of vitamin D or Patients after washout of cinacalcet); Serum calcium ≥ 2.08 mmol/L, LVH +/- cardiac fibrosis in echocardiography; Optimal fluid composition.<br>Main exclusion criteria: Unstable medical condition; Significantly impaired LV systolic function or hemodynamically effective heart valve defects; Anticipated parathyroidectomy; Scheduled kidney transplant from a living donor; Uncontrolled hyperphosphatemia; Sensitivity or intolerance to administered products; Women who are pregnant, breast feeding.<br>Eligibility criteria for study centres: both study centres perform standardized hemodialysis treatment in patients with end stage renal disease. The performance of laboratory analyses and BCM measurements is possible in both centres.<br>Eligibility criteria for individuals performing the interventions:<br>The MRIs will be performed and analysed by C.L., a radiology specialist who is blinded for patient's treatment allocation.<br>The strain echocardiograms will be performed by R.R., a specialist for internal medicine who is blinded for patient's treatment allocation.	29-30, Table 1
Interventions	11a	Cardiac MRI: at baseline and after 12 months of treatment; performed on a dialysis free day.<br>Strain echocardiogram: at screening and after 12 months of treatment.<br>Lung ultrasound: will be performed at screening.<br>Body composition monitoring: performed at the bedside for screening and in two-month intervals<br>Laboratory analyses: blood will be taken from the hemodialysis machine. Analyses will be performed in regular intervals.<br>Investigational products:<br>Patients will receive either Etelcalcetide or Alfacalcidol intravenously 3 times per week at a dosage adapted for laboratory results. Both drugs are approved medication for the patient cohort and are well tolerated.	6-12
	11b	Drug dose will be changed depending on laboratory analysis (PTH, Calcium, Phosphate).<br>Patients will discontinue the study in the case of a kidney transplant, pregnancy or severe side effects.	6-8

	11c	Study medication will be injected into the patient's blood stream via the dialysis machine by dialysis nursing staff.	11-12
	11d	There are no restrictions on calcium supplements, the dialysate calcium concentration, or the type or dose of phosphate binders prescribed. Participants randomized to Etelcalcetide can receive additional vitamin D analogs as a rescue therapy only when the investigator thinks that it is necessary to protect participant safety.	12
Outcomes	12	Primary endpoint: The change of LVMI from baseline after a year-long treatment with either etelcalcetide or alfacalcidol.<br>Secondary endpoints – cardiac structure: Difference in left atrial diameter measured by cMRI (continuous variable in mm) after a year long treatment with either drug. Change in LVMI and LAD progression in either treatment group (%). Difference in cardiac fibrosis and progression measured by cMRI (T1 mapping/relaxation) and cardiac strain (strain in different myocardial segments). Differences in cardiac function (ejection fraction - %) and wall motion abnormalities (% change)<br>Secondary endpoints – others: Changes in metabolites of the RAAS (pg/ml) using mass spectrometry ("RAAS fingerprint") under either treatment. Change from baseline serum levels of FGF23 (RU/mL) and s-klotho (pg/mL) under either drug. Change from baseline in PTH (ng/l), 25-OH-Vit-D (nmol/L) and 1,25-(OH)2-Vit-D (pg/mL), serum phosphate (mmol/l), serum calcium (mmol/l) under either treatment. Changes from baseline in proBNP (pg/ml), pre- and postdialysis TnT (ng/ml) in either medication group.	8-10
Participant timeline	13	After achieving informed consent patients will be screened, followed by a washout phase of 4 weeks (if patient is taking calcimimetic or vitamin D therapy). Following this the baseline MRI will take place. Each patient will then receive study medication for a duration of one year followed by the second MRI.	6-8

Sample size	14	Under the assumption based on published data that the median LVM/BSA of hemodialysis patients determined by cMRI is $100g/m^2$ with a SD of $25g/m^2$ and an expected treatment effect of delta LVMI of 20g/m2 roughly 2x25 patients are needed to detect this difference with 80% power at an alpha level of 0.05. Assuming a 10% attrition (drop out/loss to follow up) rate within the one year of follow up an effective sample size in the ITT analysis is 2x31 patients.	14
Recruitment	15	Patients from both dialyses centers will be asked to participate in the trial. The hemodialysis center of the Medical University of Vienna has 160 prevalent patients and the Vienna Dialysis Center has 300 prevalent patients.	6

Methods: Assignment of interventions (for controlled trials)

Allocation:

Sequence generation	16a	Randomization is performed following first MRI by a computer algorithm (www.meduniwien.ac.at/randomizer/web) stratified by residual kidney function (RKF), defined as an amount of 500ml or more urine volume per day and the center where patients are recruited. To ensure that comparison groups are of approximately the same size and balanced in each centre a block randomization (block size of 4) of anuria vs. residual renal function groups is used.	6-7
Allocation concealment mechanism	16b	A computer algorithm is used (www.meduniwien.ac.at/randomizer/web). Study staff (K.D., R.O. and dialysis nurses) is unblinded for patient's treatment allocation. (manuscript v1. 29.07.19, page 6-7)	17
Implementation	16c	K.D. is responsible for enrolment of patients, allocation of study medication using a computer program and the assignment to interventions.	
Blinding (masking)	17a	Patients are blinded for their treatment allocation. The radiologist performing the MRIs and the physician performing the echocardiograms is blinded for patient's treatment allocation.	6,8,9

Methods: Data collection, management, and analysis

Data collection methods	18a	The MedUniVienna IT infrastructure (KKS-trial database, https://www.meduniwien.ac.at/hp/kks/) is used to perform the data collection, management, quality control and analysis as state of the art with blinding and regular backup as in the many other studies we have performed in the past and are currently being performed by the study group.<br>Laboratory analyses are performed mostly by the department of laboratory under the supervision of R.M.	12,17
	18b	K.D. is in charge of promoting participant retention and complete follow-up.	17
Data management	19	The mentioned MedUniVienna IT infrastructure (KKS-trial database, https://www.meduniwien.ac.at/hp/kks/) is used for data entry, coding and storage.	12
Statistical methods	20a	A descriptive analysis will be performed and visualized with in the form of boxplots and histograms.<br>Variance homogeneity of parameters will be tested with histograms.<br>T-tests will be used for normally distributed parameters and Wilcoxon sign rank tests as a nonparametric alternative.<br>The primary endpoint (change in LVMI from baseline to final measurement) will be analyzed by Analysis of Covariance. The baseline values for each patient will be used as a covariate in the model. Furthermore, variables which will be used as stratification factors for the randomization procedure will enter the model in order to adjust for possible remaining imbalances between the groups. The secondary endpoints will also be analyzed analogously.<br>A detailed statistical analysis plan will be completed before statistical analysis.<br>Patients will be randomized to etelcalcetide or alfacalcidol after the first cMRI. Major confounders in our analysis will be dialysis vintage, fluid status and vitamin D treatment. Our approach to this will be the following: only patients with a dialysis vintage of above 3 months and below 3 years will be included in the study. BCM will be performed before enrollment and only patients who achieve their optimal dry weight through dialysis will participate in the analysis.<br>Randomization will be stratified by residual kidney function (≥ 500ml urine/day vs. ≤ 500ml urine/day) and center of recruitment.<br>We are expecting a homogeneous group of patients in both treatment arms.	13-14

	20c	An intention to treat analysis will be performed.	

Methods: Monitoring

Data monitoring	21a	A data safety monitor board (DSMB) is convened to assess the safety of treatment as well as the non-superiority of one treatment over the other based on the methods described by O'Brien & Fleming. The data safety monitor board consists of physicians who are not connected to the project or the study team and are be blinded for the patient's treatment allocation.	12
	21b	Interim analysis was performed by the board after completion of 10 cases in each treatment group (= one third of the planned study population). The Lan and DeMets extension of the O'Brien-Fleming stopping rules were applied. Based on the statistical analysis and discussion of the reported adverse events it was recommended by the DSMB to continue the study as planned.	12
Harms	22	The investigators ensure that adequate medical care is provided in any clinical situation, including emergencies. All AEs observed by the investigator or reported by subjects are properly captured in the subjects' medical records. This collection period is from the time of first dose of investigational product to 30 days after the last dose.<br>It is left to the investigator's clinical judgment to determine whether an AE is related and of sufficient severity to require the subject's removal from treatment. As defined by the International Conference on Harmonization guidelines and World Health Organization Good Clinical Practice guidelines, serious AEs are events that result in patient death, are life-threatening, require or prolong hospital stay, cause persistent or significant disability or incapacity, result in congenital anomaly or birth defect, or necessitate specific interventions. Events that are suspected unexpected serious adverse reactions (SUSARs) will be reported to the responsible ethics committee, the European Medicines Agency via the Clinical Trials Coordination Center of the Medical University of Vienna. Fatal SUSARs will be reported as soon as possible, but latest within 7 days and non-fatal SUSARs within 15 days.	13

Auditing	23	A study independent person from the Med. Univ. of Vienna serves as the study monitor. The monitor contacts and visits the investigator regularly and is allowed, on request, to have access to all source documents needed to verify the entries in the CRFs and other study related documents provided that subject confidentiality is maintained in agreement with local regulations. It is the monitor's responsibility to inspect the CRFs at regular intervals throughout the study, to verify the adherence to the study protocol and the completeness, consistency and accuracy of the data being entered on them. The monitoring standards require full verification for the presence of informed consent, adherence to the inclusion/exclusion criteria, documentation of SAEs/SADEs and the recording of the main efficacy, safety, and tolerability endpoints. At least 3 monitoring visits are scheduled.	12

Ethics and dissemination

Research ethics approval	24	Institutional ethics committee approval of the Medical University of Vienna (EK 1127/2017) and of the ethics committee of the Vienna Dialysis Center (05.09.2017) was obtained for all aspects of the study.	17
Protocol amendments	25	Important protocol modifications will be reported to relevant parties (Ethics committee, Federal Office for Safety in Health Care, Austrian Agency for Health and Food Safety)	14,17
Consent or assent	26a	Patients will be informed about the study and asked to participate by the investigators who are also the patient’s treating medical doctors (i.e. K.D., R.O., M.L.). Furthermore patients will receive written information about the study. Patients have to give oral and written consent in order to participate.	6,17
Confidentiality	27	Confidentiality of subjects in reports/publications will be guaranteed. The obtained data in the database is de-personalized (pseudonimized).	17
Declaration of interests	28	There are no financial and other competing interests for principal investigators for the overall trial and each study site.	16,17

Access to data	29	Only members of the research team will have access to the database.	12,17
Ancillary and post-trial care	30	An insurance for each patient enrolled in this study is taken out. The company Zürich provides the insurance cover.	17
Dissemination policy	31a	The findings of this study will be published by the investigators in a scientific journal and presented at scientific meetings. The manuscript will be circulated to all co-investigators before submission. Confidentiality of subjects in reports/publications will be guaranteed. Rights to an authorship are according to GCP and Good Scientific Practice (GSP) of the Medical University of Vienna. It is planned that Dr. Katharina Dörr, will be the primary author of the manuscript.	17

Appendices

Informed consent materials	32	Each patient will receive an informed consent form in German language previously approved by the Ethics committee.	

*It is strongly recommended that this checklist be read in conjunction with the SPIRIT 2013 Explanation & Elaboration for important clarification on the items. Amendments to the protocol should be tracked and dated. The SPIRIT checklist is copyrighted by the SPIRIT Group under the Creative Commons "Attribution-NonCommercial-NoDerivs 3.0 Unported" license.
